# Modelling chronic malnutrition in Zambia: A Bayesian distributional regression approach

**DOI:** 10.1371/journal.pone.0255073

**Published:** 2021-08-04

**Authors:** Given Moonga, Stephan Böse-O’Reilly, Ursula Berger, Kenneth Harttgen, Charles Michelo, Dennis Nowak, Uwe Siebert, John Yabe, Johannes Seiler

**Affiliations:** 1 Center for International Health, Ludwig Maximilian University of Munich, Munich, Germany; 2 Department of Public Health, Health Services Research and Health Technology Assessment, UMIT—University for Health Sciences, Medical Informatics and Technology, Hall in Tirol, Austria; 3 Department of Epidemiology and Biostatistics, University of Zambia, Lusaka, Zambia; 4 Institute and Outpatient Clinic for Occupational, Social and Environmental Medicine, Clinical Centre of the Ludwig Maximilian University of Munich, Munich, Germany; 5 Institute for medical Information Processing, Biometry, and Epidemiology, Ludwig Maximilian University of Munich, Munich, Germany; 6 Department of Humanities, Social and Political Sciences, ETH Zurich, Zurich, Switzerland; 7 School of Veterinary Medicine, University of Zambia, Lusaka, Zambia; 8 Department of Statistics, University of Innsbruck, Innsbruck, Austria; National Taiwan University, TAIWAN

## Abstract

**Background:**

The burden of child under-nutrition still remains a global challenge, with greater severity being faced by low- and middle-income countries, despite the strategies in the Sustainable Development Goals (SDGs). Globally, malnutrition is the one of the most important risk factors associated with illness and death, affecting hundreds of millions of pregnant women and young children. Sub-Saharan Africa is one of the regions in the world struggling with the burden of chronic malnutrition. The 2018 Zambia Demographic and Health Survey (ZDHS) report estimated that 35% of the children under five years of age are stunted. The objective of this study was to analyse the distribution, and associated factors of stunting in Zambia.

**Methods:**

We analysed the relationships between socio-economic, and remote sensed characteristics and anthropometric outcomes in under five children, using Bayesian distributional regression. Georeferenced data was available for 25,852 children from two waves of the ZDHS, 31% observation were from the 2007 and 69% were from the 2013/14. We assessed the linear, non-linear and spatial effects of covariates on the height-for-age z-score.

**Results:**

Stunting decreased between 2007 and 2013/14 from a mean z-score of 1.59 (credible interval (CI): -1.63; -1.55) to -1.47 (CI: -1.49; -1.44). We found a strong non-linear relationship for the education of the mother and the wealth of the household on the height-for-age z-score. Moreover, increasing levels of maternal education above the eighth grade were associated with a reduced variation of stunting. Our study finds that remote sensed covariates alone explain little of the variation of the height-for-age z-score, which highlights the importance to collect socio-economic characteristics, and to control for socio-economic characteristics of the individual and the household.

**Conclusions:**

While stunting still remains unacceptably high in Zambia with remarkable regional inequalities, the decline is lagging behind goal two of the SDGs. This emphasises the need for policies that help to reduce the share of chronic malnourished children within Zambia.

## Introduction

The burden of child malnutrition still remains a global challenge, with greater severity being faced by low-and middle-income countries [1–3]. Globally, malnutrition is amongst the most important risk factors associated with illness and death, affecting hundreds of millions of pregnant women and young children [3–6]. Stunting in early childhood is strongly associated with numerous short-term and long-term consequences, including increased childhood morbidity and mortality, delayed growth and motor development and long-term educational and economic consequences later in life [7]. Undernourishment causes children to start life at mentally suboptimal levels [8].

Assessment of childhood malnutrition commonly relies on standard anthropometric measures for insufficient height-for-age (stunting) indicating chronic undernutrition, insufficient weight-for-height (wasting), indicating acute undernutrition; and insufficient weight-for-age (underweight), an indicator commonly used to asses, both, chronic, and acute undernutrition [9, 10].

Anthropometric measurements are practical techniques for assessing children’s growth patterns during the first years of life. The measurements also provide useful insights into the nutrition and health situation of entire population groups. Anthropometric indicators are less accurate than clinical and biochemical techniques in assessing individual nutritional status. However in resources limited settings, the measurements are a useful screening tool to identify individuals at risk of undernutrition, who can later be referred to subsequent possible confirmatory investigation [11].

It is estimated that globally 52 million children under-five years of age are wasted, 17 million are severely wasted and 155 million are stunted. Around 45% of deaths among children under-five years of age, most of which occur in the sub-Saharan Africa are linked to undernutrition [3, 9]. It is also estimated that four out of ten children under the age of five in Zambia are stunted [12]. This paper will therefore focus on childhood stunting in Zambia.

Global prevalence of stunting in children younger than five years declined during the past two decades, but still remain unacceptably high in South Asia and sub-Saharan Africa regions [5]. If current trends remain unchecked, projections indicate that 127 million children under five years of age will be stunted in 2025 [1]. There is therefore need to heighten various interventions in these affected region and to investigate possible area specific determinants of stunting.

There are already fairly well documented perspectives on determinants of malnutrition. The treatise on these determinants mainly relies on the United Nations Children’s Fund (UNICEF) conceptual framework on malnutrition which has evolved over time as more knowledge and evidence on the causes, consequences and impacts of undernutrition is generated. The framework distinguishes between immediate, intermediate and underlying determinants of malnutrition [5, 13–15].

The immediate causes of undernutrition include inadequate dietary intake and disease, while the underlying causes could include household food insecurity, inadequate care and feeding practices for children, unhealthy household and surrounding environments, and inaccessible and often inadequate health care. Basic causes of poor nutrition encompasses the societal structures and processes that neglect human rights and perpetuate poverty, constraints faced by populations to essential resources [13].

Several studies done within sub-Saharan Africa investigated determinants such as the mother’s level of education, income levels and these factors have been linked to malnutrition [9, 12, 16, 17]. The source of the drinking water, the wealth of the household, the area of residence, age of the child, the sex of the child, the breastfeeding duration, the age of the mother has also been investigated and were observed to be significant correlates of stunting [12, 18]. Within Zambia, stunting was observed to be more likely among children of less educated mothers (45%) and those from the poorest households (47%) [19]. The determinates of malnutrition are related to each other and the differences and direction between these levels of determinism as indicated in the UNICEF framework are often not discrete but in reality related. As discussed by Kandala [17] for example, the mother’s level of education might be influencing child care practises- an intermediate determinant—and the resources available to the household—an underlying determinant.

**Fig 1:**
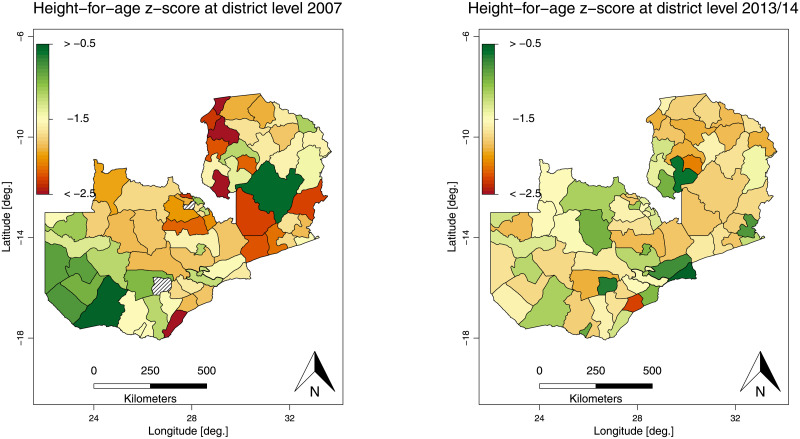
Levels of stunting over time in Zambia. The panel shows the average height-for-age z-score at district level for 2007 (left) and 2013/14 (right) of Zambia. *Source*: Demographic and Health Surveys (data) and Database of Global Administrative Areas (boundary information); calculation by authors. The shapefile used to create these maps is republished from [54] under CC BY license, with permission from Robert J. Hijmans, original copyright [2021].

Previous studies elsewhere have observed that stunting tends to show regional variation [4, 9, 16]. We see this trend in Zambia as well, where the decline of stunting has been only gradual and unacceptable, with higher prevalence in Northern province where 50% of the children being stunted, and stunting being less common in Lusaka, Copperbelt, and Western provinces where 36% of children are stunted [19]. We see this regional variation of stunting in Fig 1 which shows stunting in Zambia in the 2007 and 2013/14 waves of the Zambian Demographic and Health Surveys (ZDHS). The ZDHS is a national-wide survey which is representative at a sub-national level and contains information on trends in fertility, childhood mortality, use of family planning methods, and maternal and child health indicators including HIV and AIDS [19]. The figure shows the height-for-age z-score, with Western province better than Northern province for the 2007 wave. We see a slight difference in 2013/14 as stunting seemed to get worse in parts of the Western province.

Much of the work done on the determinants of stunting in Zambia, have considered socio-economic characteristics and have assessed the linear effects of these determinants on the conditional mean [12, 20], using models specifications such as; linear models, generalized linear models (GLMs) and generalized additive models (GAMs) [21]. These aproaches are useful and have the advantage of being easy to estimate and to interpret. However, they may risk model misspecification and draw inaccurate estimates, when heterogeneity is present, or when extreme values in the response are present and when a linear relationship is not plausible. In the analysis of certain outcomes, like stunting for example, the interest is not only in the conditional mean, but also in extreme values (height-for-age z-scores), or other parameters of the response. Quantile regression is one possibility to model beyond the conditional mean, with the interest to show variation of the outcome at a quantile level, without making any assumptions of the response distribution. This method for example has been applied in child malnutrition studies [16]. However, distributional regression offers advantage over quantile regression, as it provides the possibility to characterize the complete probabilistic distribution of the response in one joint model [21, 22]. Moreover, distributional regression is more efficient, if prior knowledge on specific aspects of the response distribution is available, or can be estimated [23]. Furthermore, the characterization of the whole distribution of the response is more informative.

The study by Kandala [24] which focused on stunting in sub-Saharan countries found that there are distinct spatial patterns of malnutrition that are not explained by the socio-economic determinants or other well-known correlates alone [16, 25]. As such, our study includes spatial covariates, since we aim at investigating spatial differences of stunting in Zambia at sub-district level while jointly analysing socio-economic and environmental characteristics.

The following three reasons make our study novel compared to previous work [18]. Firstly, we jointly analysed remote sensed data and socio-economic covariates at sub-national level. This is made possible due to availability of georeferenced data at the primary sampling unit a household pertains to in the recent two ZDHS datasets. The Demographic and Health Surveys rely in most cases on a two-stage survey, and the primary sampling units corresponds to the enumeration areas from the most recent completed census that has been selected. Georeferenced data is important as it generates more specific information which can facilitate targeted interventions. Secondly, we used Bayesian distributional regression which allows us to model all parameters of the underlying response distribution. Lastly, we used two waves of the demographic health surveys to control for spatio temporal interactions. Therefore, this study demonstrates small area variation in stunting in Zambia and analyse possible inequalities and deprivation at the sub-district level.

## Data sources

### Socio-economic and georeferenced covariates

We used data from the 2007 and 2013/14 ZDHS. The ZDHS is a national-wide survey which is representative at a sub-national level and contains information on trends in fertility, childhood mortality, use of family planning methods, and maternal and child health indicators including HIV and AIDS. For these population health indicators, data is collected for women aged 15–49, men aged 15–59 and children below five years of age [19].

The ZDHS provide besides information on the district a household pertains to, also information about the geolocation of the primary sampling unit a household belongs to, and from which the data was collected. The location of the primary sampling unit is the spatial information used in the empirical analysis. During data processing, GPS coordinates are displaced to ensure that respondent confidentiality is maintained. The displacement is randomly applied so that rural points contain a minimum of 0 and a maximum of 5 km of positional error. Urban points contain a minimum of 0 and a maximum of 2 km of error. A further 1% of the rural sample points are offset a minimum of 0 and a maximum of 10 km [26].

Demographic Health Surveys have documented weakness for estimation of individual anthropometric measurements. Potential threats to high data quality may occur across various research stages, from survey design to data analysis. There is also often a substantial amount of missing or implausible anthropometric data across surveys [27].

Furthermore, there is caution over the use of stunting as an individual classifier in epidemiologic research or its interpretation as a clinically meaningful health outcome. Stunting should be used as originally designed to be from its original use as a population level indicator of community well-being [28], as it reflects past health and nutrition conditions; and an indication of socio-economic development of a country [1].

Despite the above highlighted limitations of DHS and anthropometric indicators, they remain useful national wide measurements that can be used to estimate child health. Moreover, in general anthropometric measures are a good indicator for planning as they can provide a lot of information to policy makers to answer, how, where and which type of intervention would be favourable in specific settings.

### Socio-economic and spatial determinants

The effects of socio-economic factors, such as the education of the mother, household size, wealth of the household on the health status of children are well documented [12, 29]. We calculated an index representing the wealth of the household based on the household’s assets using Principal Components Analysis (PCA) following Filmer and Pritchett, and Sahn [30, 31]. Previous studies have shown that household wealth status was a predictor of childhood malnutrition. Children from poor households are more likely to be stunted than those from richer households [29].

In our analysis we investigated the impact of different socio-economic factors, which impact on height-for age Z-score has been discussed in literature. Table 1 gives an overview and the according references.

**Table 1:** Included covariates, their source, and effect on the height-for-age z-score found in the literature.

Covariate	Used data source	Effect on stunting found in literature	Reference
Asset Index	DHS	Household wealth inequality associated with childhood stunting	[29]
Age mother at birth	DHS	Increasing non-linearly	[16]
Age child	DHS	Decreasing non-linearly	[17]
Birth order	DHS	Being born forth or higher significantly more stunted	[16]
Breastfeeding duration	DHS	Breastfeeding interval ≤ associated with low level of stunting	[25]
Education mother	DHS	Stunting improves non-linearly with the educational level	[32]
Household size	DHS	Increases linearly	[12]
Mothers’ BMI	DHS	U-shape relationship with childhood stunting	[17]
Number of vaccinations	DHS	Lower levels of stunting when fully vaccinated	[25]
Drought severity index	See Table 2	Not further specified	[33]
Malaria incidence	See Table 2	No clear pattern	[34]
Population density	See Table 2	Not further specified	[33]

### Remote sensed covariates

We obtained remote sensed data on drought severity, malaria incidence, and population density. The description, and source to these data sets is provided in Table 2.

**Table 2:** Source of remote sensed covariates.

Covariate	Description	Source	Reference
Drought index	scPDSI CRU4.03	Climate Research Unit	[36, 37]
Malaria incidence	Plasmodium falciparum incidence	Malaria Atlas Project	[35]
Population density	Number of people per km^2^	Socioeconomic Data and Applications Center	[38]

For example, the malaria incidence data was obtained from the Malaria Atlas Project (MAP). The project collects malaria data on malaria cases reported by surveillance systems, nationally representative cross-sectional surveys of parasite rate, and satellite imagery capturing global environmental conditions that influence malaria transmission [35].

## Methodology

We assessed the relationships between socio-economic and remote sensed characteristics and anthropometric outcomes using the Bayesian Distributional Regression (BDR). BDR models all parameters of the response distribution based on structured additive predictors and allows to incorporate for example, non-linear effects of metric covariates, spatial effects, or varying effects. Applications of structured additive regression models to topics in Global Public Health are found in several publications [39–42]. This approach permits us to fully analyse the whole distribution [41, 43] and our analysis was not restricted to assessing the conditional mean of the height-for-age z-score. Instead suspected heterogeneity across socio-economic and georeferenced factors and the anthropometric measure can be directly captured. In the context of growth failures this is of particular importance, as previous studies highlighted high levels of heterogeneity related to growth failures [33].

### Bayesian distributional regression

**Fig 2:**
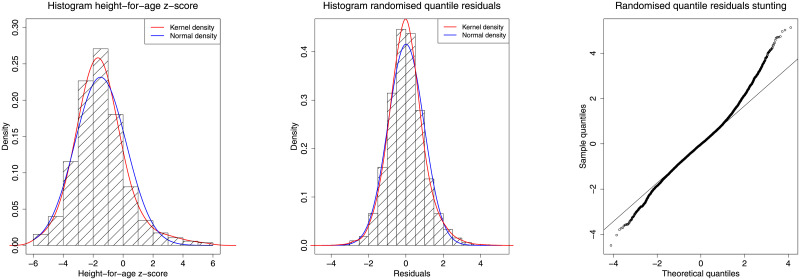
Histogram of the response and histogram and QQ-plot of the randomised quantile residuals. The left-hand panel shows the histogram and kernel density estimates of the height-for-age z-score, the middle panel shows the histogram of the randomised quantile residuals together with the normal density estimates, and the right-hand panels depicts the QQ-plot of the randomised quantile residuals. *Source*: Demographic and Health Surveys (data); calculation by authors.

Relying on Bayesian distributional regression requires to specify the distribution of the response variable. Assuming the response distribution to be Gaussian permits to model besides the conditional mean also the variance or standard deviation of the response variable. Graphical analysis using amongst others randomised quantile residuals [44] strengthens that a Gaussian model is plausible. See also Fig 2, for more details. In the left- hand panel of Fig 2 the histogram of the height-for-age z-score together with the underlying density illustrates why the normal distribution seems to be an appropriate choice. This is further confirmed in the second and third panel, where the histogram of the quantile residuals including the underlying kernel density estimate, respectively, the QQ-plot of the randomised quantile residuals are shown.

Assuming the response distribution of the height-for-age z-score to be Gaussian, both the mean *μ* and the standard deviation *σ* are related to a structured additive predictor. Accordingly, the $z-{\text{score}}_{is}∼N\left(\mu_{is},\sigma_{i}\right)$ can be specified as Gaussian response, with *i* = 1, …, *I* being the number of children, and *s* = 1, …, *S* be the location of the primary sampling unit the child pertains to. Using the same notation as in the methodology manual of BayesX the regression model can be written as follows [45]:

$$\begin{array}{l} \mu =h_{\mu }(\eta_{\mu })=\eta_{\mu },\\ \sigma =h_{\sigma }(\eta_{\sigma })=\text{exp}(\eta_{\sigma }). \end{array}$$ (1)

Here both parameters of the normal distribution the mean *μ* and the standard deviation *σ* are related to the set of covariates specified further in Tables 1 and 2. Accordingly, the response functions *h*_*μ*_, and *h*_*σ*_ link the two parameters to their structured additive predictors which is specified as follows:

$$\begin{array}{ll} \eta_{\mu }= & f_{1}(Asset\;index)+f_{2}(Birth\;order,\;Age\;mother\;at\;birth)+\\ & f_{3}(Age\;child,\;Breastfeeding\;duration)+f_{4}(Education\;mother)+\\ & f_{5}(Household\;size)+f_{6}(BMI\;mother)+\\ & f_{7}(Number\;of\;vaccinations)+f_{8}(Drought\;severity\;index)+\\ & f_{9}(Malaria\;incidence)+f_{10}(\text{log}(1+Population\;density))\\ & f_{11}(Spatial,\;Time)+x^{\prime }\beta ,\\ \eta_{\sigma }= & f_{1}(Asset\;index)+f_{2}(Birth\;order,\;Age\;mother\;at\;birth)+\\ & f_{3}(Age\;child,\;Breastfeeding\;duration)+f_{4}(Education\;mother)+\\ & f_{5}(Household\;size)+f_{6}(BMI\;mother)+\\ & f_{7}(Number\;of\;vaccinations)+f_{8}(Drought\;severity\;index)+\\ & f_{9}(Malaria\;incidence)+f_{10}(\text{log}(1+Population\;density))\\ & f_{11}(Spatial,\;Time)+x^{\prime }\beta , \end{array}$$ (2)

where *f*_1_(⋅) to *f*_10_(⋅) are potential non-linear effects of socio-economic and remote sensed covariates approximated using Bayesian penalised Splines (P-Splines) first described by Lang and Brezger [46]. Bayesian P-Splines are based on P-Splines as introduced by Eilers and Marx [47], and use an approach based on basis functions. As smoothness priors of the unknown regression parameters ***β***_*j*_ a random walk prior of the form $\beta_{j}|\gamma_{j}^{2}\propto \text{exp}(-\frac{1}{2\gamma_{j}^{2}}\beta_{j}^{\prime }K_{j}\beta_{j})$ is specified, with $\gamma_{j}^{2}$ being random variance parameters and ***K***_*j*_ is an appropriate penalty matrix, see also Lang and Brezger [46] for an elaborate discussion. Relying on Bayesian P-Splines allows to incorporate different model terms such as non-linear effects for continuous variables, varying coefficients [48], or spatial effect. In addition, Bayesian P-Splines allow to decompose the predictor additively and are known for having good mixing properties. See S1 to S8 Figs in the Supporting Information for convergence diagnosctics of the sampling paths of the parameters included in the final model that is based on a Markov chain Monte Carlo (MCMC) algorithm. *f*_11_(⋅) is the spatio-temporal effect included in the model to account for unexplained heterogeneity by incorporating a Markov random field prior [46]. In more detail, following Lang and Brezger [46], the basic Markov random field prior for the regression coefficients *β*_s_ of the spatially correlated effect *f*_*s*_ is defined as follows: $\beta_{\text{s}}|\beta_{{\text{s}}^{\prime }},\text{s}\neq {\text{s}}^{\prime }\sim N(\frac{1}{N_{\text{s}}}\sum_{{\text{s}}^{\prime }\in \delta_{\text{s}}}\beta_{{\text{s}}^{\prime }},\frac{\tau_{\text{s}}^{2}}{N_{\text{s}}})$. Where *N*_s_ is the number of neighbouring sites of location s, s′ belongs to the set of neighbouring sites *δ*_s_ of location s, and $\tau_{\text{s}}^{2}$ being the spatially adaptive variance parameteres [47]. Incorporating the spatial effect on a Markov random field proposal allows to account for the remaining heterogeneity that is not explained by the included covariates. See also Chapter 4 of the methodology manual of **BayesX** [45], and Seiler and colleagues [42] for a more elaborate discussion on the incorporation of the spatio- temporal effect based on a Markov random field prior. This variables are further specified in Table 1. ***x***′ ***β*** subsumes the vector of included effect coded categorical covariates that are the gender, the place of living, and the survey wave. For an illustration of effect coding see for example Fahrmeier and colleagues [49]. From a Bayesian perspective the categorical variables are considered to be random variables for which a diffuse prior of the form *p*(*γ*_*j*_) ∝ const is assigned [46].

### Model selection

The fit of the models are compared by relying on the Deviance Information Criterion (DIC) [50] and Widely Applicable Information Criterion (WAIC) [51], and are summarised in Table 3. As a rule of thumb can be seen that the model with the lowest value describes the data best. We specified six distinct models, aiming to identify the importance of, for instance, socio-economic or georeferenced factors. In more detail, the differences between these models are summarised in Table 4.

**Table 3:** Estimation results: DIC and WAIC.

Model	DIC	WAIC
Model 1	95588.2	95550.7
Model 2	91027.1	91566.4
Model 3	91375.7	91884.0
Model 4	96263.1	96541.9
**Model 5**	**90992.4**	**91527.7**
Model 6	91012.7	91543.3

Values of the DIC and the WAIC for different model specifications. *Source*: DHS; calculation by authors.

**Table 4:** Specification of estimated models.

Model term	Model 1	Model 2	Model 3	Model 4	Model 5	Model 6
***η***_***μ***_	yes (y)	y	y	y	y	y
***η***_***σ***_	n (n)	y	y	y	y	y
***x***′ ***β***	y	y	y	y	y	y
*f*_1_(Asset index)	y	y	y	n	y	y
*f*_2_(Birth order, Age mother at birth)	y ***η***_***μ***_, n ***η***_***σ***_	y ***η***_***μ***_, n ***η***_***σ***_	y ***η***_***μ***_, n ***η***_***σ***_	n	y ***η***_***μ***_, n ***η***_***σ***_	y ***η***_***μ***_, n ***η***_***σ***_
*f*_3_(Age child, Breastfeeding duration)	y ***η***_***μ***_, n ***η***_***σ***_	y ***η***_***μ***_, n ***η***_***σ***_	y ***η***_***μ***_, n ***η***_***σ***_	n	y ***η***_***μ***_, n ***η***_***σ***_	y ***η***_***μ***_, n ***η***_***σ***_
*f*_4_(Education mother)	y	y	y	n	y	y
*f*_5_(Household size)	y	y	y	n	y	y
*f*_6_(BMI mother)	y	y	y	n	y	y
*f*_7_(Number of vaccinations)	y	y	y	n	y	y
*f*_8_(Drought severity index)	y	y	y	n	y	y
*f*_9_(Malaria incidence)	y	y	y	n	y	y
*f*_10_(log(1 + Population density))	y	y	y	n	y	n
*f*_11_(Spatial, Time)	y	y	y	n	y	y

Table of specified models indicating the differences between models and the included model terms and covariates. Note that after evaluating the sampling paths of the resulting Markov chains of the MCMC simulations, in ***η***_***σ***_ *f*_2_(⋅) and *f*_3_(⋅) had to be omitted due bad mixing.

## Results

In the following Section we will discuss the results of Model 5, omitting insignificant terms, as Model 5 has both the lowest DIC and WAIC. Result of the included covariates are however, similar throughout all specifications.

### Descriptive analysis

Table 5 shows the baseline characteristics of selected covariates in the population between the two ZDHS survey of 2007 and 2013/14, and remote sensed data aggregates for these waves.

**Table 5:** Descriptive statistics covariates.

	2007 ZDHS		2013/14 ZDHS	
**Response**	Mean (95% CI)	*n*	Mean (95% CI)	*n*
Stunting	-1.59 (-1.63; -1.55)	7,936	-1.47 CI (-1.49; -1.44)	17,916
**Covariates**	Mean (95% CI), %	SD	Mean (95% CI), %	SD
Proportion of male children (= 1)	49.62%		50.08%	
Age children in months	29.14 (28.76; 29.51)	17.03	29.84 (29.58; 30.09)	17.20
Breastfeeding duration in months	16.22 (16.07; 16.38)	7.04	15.69 (15.58; 15.79)	7.11
Birth order within household	2.99 (2.93; 3.04)	2.38	2.88 (2.84; 2.91)	2.37
Number of vaccinations	5.64 (5.59; 5.69)	2.40	7.45 (7.42; 7.49)	2.17
Age mother at birth in years	24.30 (24.15; 24.45)	6.82	24.08 (23.98; 24.18)	6.94
BMI mother	22.35 (22.27; 22.43)	3.42	22.57 (22.51; 22.62)	3.75
Years of education mother	7.18 (7.10; 7.26)	3.53	7.81 (7.76; 7.87)	3.64
Urban place of living (= 1)	38.73%		43.01%	
Size of the household	6.27 (6.21; 6.32)	2.49	6.60 (6.56; 6.64)	2.77
Asset index deviation regional mean	0.00 (-0.02; 0.02)	0.88	-0.01 (-0.02; 0.01)	0.88
Malaria incidence	0.26 (0.25; 0.26)	0.11	0.20 (0.20; 0.20)	0.12
Population density	260.07 (241.79; 278.36)	831.22	321.87 (305.78; 337.96)	1098.58
Drought severity index	-0.59 (-0.60; -0.58)	0.66	0.37 (0.36; 0.39)	0.91

Descriptive statistics of categorical and continuous covariates. Note that the Drought severity index corresponds to the self-calibrating Palmer Drought Severity Index (scPDSI). *Source*: DHS and other sources (see Table 2 for detailed information); calculations by authors.

Data was available for 25,852 children from the two waves, 31% observation were from the 2007 and 69% were from the 2013/14 ZDHS. Levels of stunting decreased between 2007 and 13/14 from a mean z-scores of -1.59 CI(-1.63; -1.55) to -1.47 CI(-1.49; -1.44). The breastfeeding duration declined from 16.22 to 15.69. There was a notable increase in the number of received vaccinations by children from 5.6 to 7.5 vaccinations. There was a slight increase in the number of years the mother spent in school from 7.2 to 7.8. Malaria incidence rates (plasmodium falciparum incidence) declined from 26% to 20%. Night-time light increased from 2.75, to 3.72 (observed values were log transformed), a possible indication of increase in urbanisation. Night-time light was highly correlated (*ρ* = 0.73) to population density as such it was omitted in subsequent analysis.

High disparities in the height-for-age z-score have been observed at the district and provincial level within Zambia. There was a drift in the spatial pattern of malnutrition in the 2013/14 wave compared to the previous survey, indicating a general improvement. See also Fig 1 for a more detailed, descriptive, analysis of the spatial patterns of the height-for-age z-score within Zambia.

We observed that in the 2007 wave, stunting was lowest in the Western and Muchinga province. In the Southern province generally, low values were also observed, except for the Sinazongwe district. For Eastern province, Nyimba, Katete, Petauke and Lundazi districts had high levels. In the Luapula province, high levels of stunting were observed in the districts of Milenge, Mwense, Kawambwa, Nchelenge and Chiengi. Stunting was severe in some parts of the Copperbelt province which is predominantly a mining region and the Northern province. Central province had moderate levels, except for Serenje district.

### Linear effects

With respect to the linear effects, Table 6 shows the effect of the gender and the area of residence on the posterior mean of the response variable. Considering the posterior mean of the height-for-age z-score of -1.70, boys were found to more stunted compared to girls. Stunting was also found to be higher in children from rural households compared to urban areas. Two patterns well documented in the literature for other countries and also Zambia [12, 17, 52].

**Table 6:** Estimation results: Linear effects of Model 5.

	Covariate	Posterior mean	95% Credible interval
***η***_***μ***_	Intercept	-1.70	-1.84; -1.57
	Boys	-0.10	-0.12; -0.08
	Urban	0.04	0.00; 0.07
	Wave ZDHS 2007	-0.07	-0.13; -0.01
***η***_***σ***_	Intercept	0.23	0.17; 0.29
	Boys	-0.00	-0.01; 0.01
	Urban	0.01	-0.01; 0.04
	Wave ZDHS 2007	0.05	0.01; 0.09

Results of linear covariates included in the Model 5. *Source*: DHS; calculation by authors.

### Socio-economic characteristics

**Fig 3:**
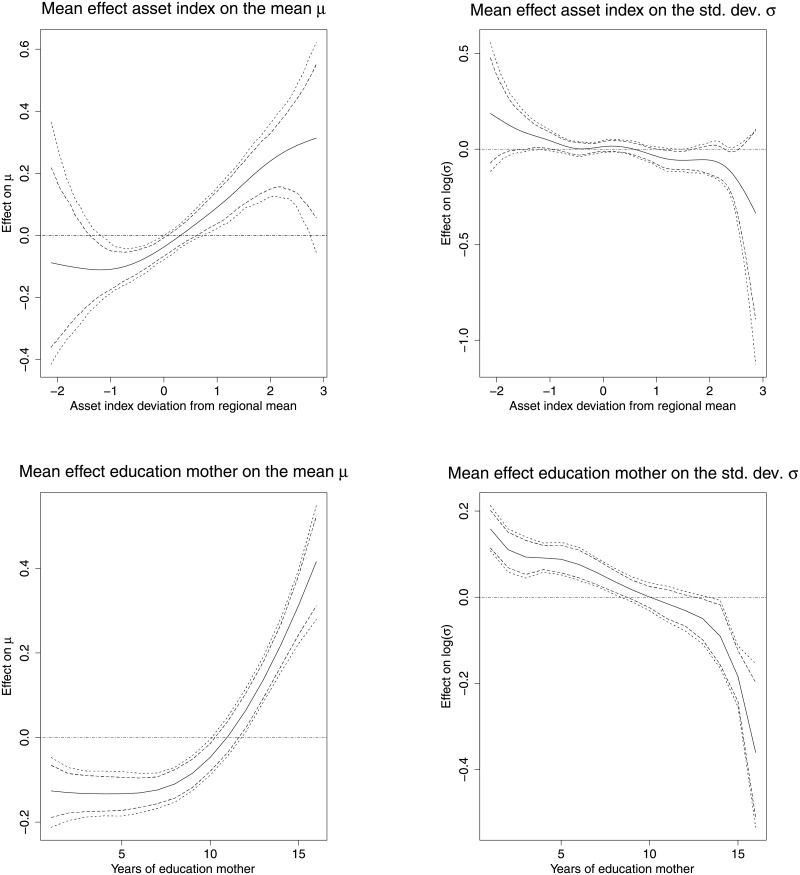
Non-linear effects of the asset index and the years of education. The Figure depicts the mean effects on the mean *μ* (left), and the standard deviation *σ* (right) together with 80 per cent and 95 per cent simultaneous credible intervals for the asset index (top), and the years of education of the mother (bottom). *Source*: Demographic and Health Surveys (data); calculation by authors.

Fig 3 shows the non-linear effect of the asset index, and the years of education of the mother on the mean *μ* and standard deviation *σ* of the response variable. Undernutrition has been associated to poverty [4], we observed that children living in poor household showed worse outcomes compare to children living in wealthier households, i.e the z-score is linearly increasing with increasing asset index. The effect of the asset index on the standard deviation does not notably vary across the range of the asset index, indicating a homogenous effect of wealth.

The bottom panel of Fig 3 shows that an increase in the level of education of the mother above eight years of education is associated with an appreciable increase in the height-for-age z-score. Notably also is that, there is not much difference in this effect for mother’s education less than 8 years. This entails that primary school education does not improve the nutrition outcome of the children as much. Above eight years of schooling, we see clearly that increase in the years has positive effect on the z-score. Moreover, the variation in the height-for-age z-score is higher with less years of schooling, whereas the variation gradually decreases with increasing levels of education of the mother.

**Fig 4:**
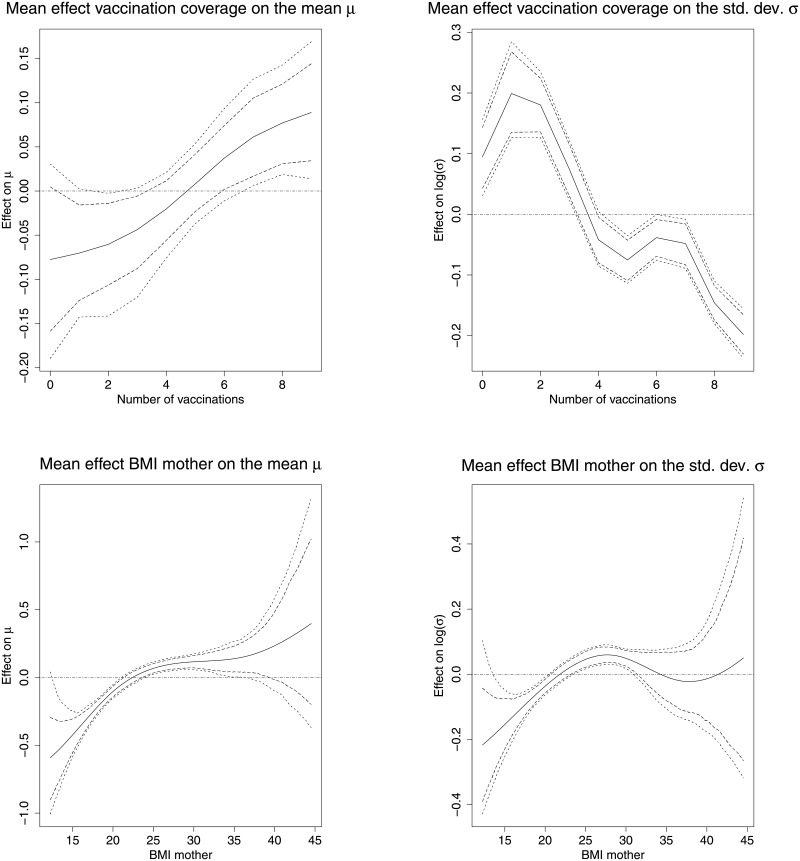
Non-linear effects of the vaccination coverage and the BMI of the mother. The Figure depicts the mean effects on the mean *μ* (left), and the standard deviation *σ* (right) together with 80 per cent and 95 per cent simultaneous credible intervals for the vaccination coverage (top), and the BMI of the mother (bottom). *Source*: Demographic and Health Surveys (data); calculation by authors.

Fig 4 shows the non-linear effect of the number of vaccinations the child received and mother’s BMI on the mean and the standard deviation z- score. The top graph shows that there was a positive effect on the mean z-score with increase in the number of vaccinations the child received. There is also greater variation in stunting levels among children who received less than 2 vaccinations.

Low values of the mothers BMI are negatively associated with the height-for-age z-score of the child, while for increasing values of the BMI also an increase in the posterior mean of the z/score can be observed. For values above 40 for the BMI of their mother the results are inconclusive indicated by the widening of the credible intervals. Low values of the BMI of the mother are associated with less variation compared to high values.

**Fig 5:**
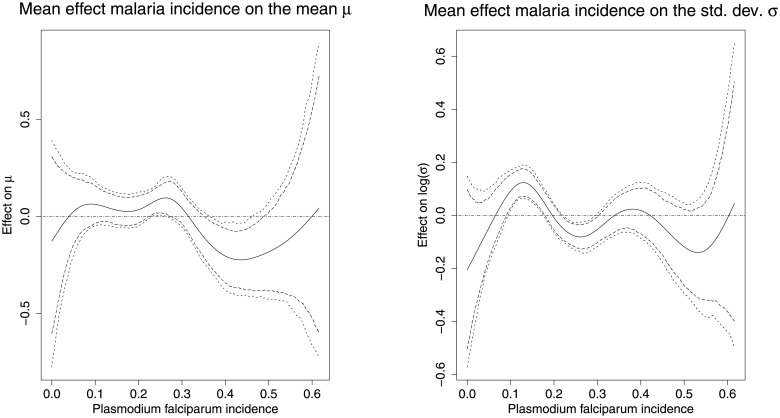
Non-linear effects of the malaria incidence. The Figure depicts the mean effects on the mean *μ* (left), and the standard deviation *σ* (right) together with 80 per cent and 95 per cent simultaneous credible intervals for the malaria incidence. *Source*: Demographic and Health Surveys (data); calculation by authors.

Fig 5 shows that increasing malaria incidence about 0.3 had a negative effect on the z-score, however we do not see any meaningful differences in the standard deviation over the spectrum the malaria incidences.

**Fig 6:**
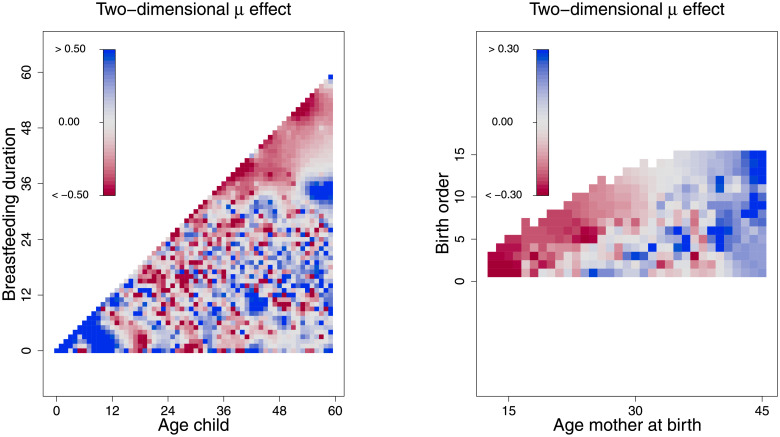
Smooth effects of the interaction of the age of the child and breastfeeding duration, and interaction the age of the mother and the birth order. The Figure depicts the mean effects on the mean *μ* for the interactions of the age of the child and breastfeeding duration, and the interaction the age of the mother and the birth order, respectively. *Source*: Demographic and Health Surveys (data); calculation by authors.

Due to the high correlation of breastfeeding and age of the child, an interaction between these two variables can be presumed for which one has to account for. Fig 6 shows that children below 12 months of age who were breastfeed, were not malnourished. Accordingly, malnutrition mostly seems to be a process that comes to effect as children grow. Stunting was high for children above 36 months of age and who were breastfeed. On the right side, the figure shows that stunting was low in children whose mothers were around 30 years and with respect to birth order, which emphasizes that especially children of very young mothers are those most vulnerable.

### Georeferenced characteristics

**Fig 7:**
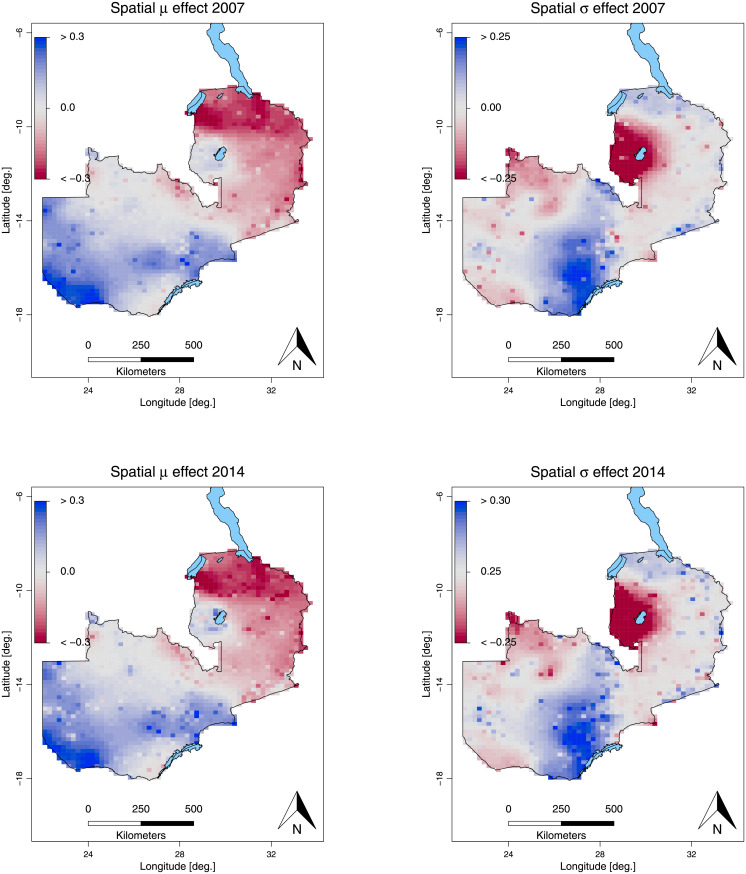
Smooth spatial effect. The Figure depicts the mean spatial effect of the mean *μ* (left), and the standard deviation *σ* (right) for the year 2007 (left) and the year 2013/14 (right). *Source*: Demographic and Health Surveys (data) and Database of Global Administrative Areas (boundary information); calculation by authors. The shapefile used to create these maps is republished from [54] under CC BY license, with permission from Robert J. Hijmans, original copyright [2021].

Fig 7 emphasizes the pronounced north and south pattern after adjusting for all the other variables, in particular after adjusting for wealth and rurality which was already described in the descriptive analysis. The highest variation in the height-for-age z-score was also observed in north (Luapula province) in both waves as can be seen on the right side of the figure.

## Discussion & conclusion

Using the two waves of the ZDHS, we modelled the height-for-age z-score by using socio-economic and remote sensed information. To analyse the whole distribution and not just focusing on the conditional mean, we used a Bayesian distributional regression approach accounting for heterogeneity as well.

Using Bayesian distributional regression, we assessed the relationship of socio-economic, and remote sensed covariates and stunting. Bayesian distributional regression, presents an advantage in terms of model flexibility allowing to incorporate, amongst others, non-linear effects and spatial effects. This however comes also with the drawback of data intensity and computational complexity.

Remote sensed techniques can be useful for future research on community health assessment as these techniques provide an advantage to take measurements quickly for remote and hard to reach areas. The data also enable to make meaningful analyses at sub-national levels which can improve targeting of interventions due to high levels of geographic specificity [26, 33], however they do not give a full picture. Therefore, it is important to account for other covariates such as socio-economic characteristics at the individual or household level.

When relying on remote sensed information to asses anthropometric measures or biophysical developments, great caution should be taken with respect to data quality. Our study finds that remote sensed covariates alone explain little of the variation of the response, this emphasizes the need to control also for socio-economic characteristics. We find that the combination of remote sensed data and socio-economic characteristics explain more of the variation of the response, compared to solely focusing on one of the two sources of explanatory variables. In addition this also highlights the strong influence of socio-economic covariates or can be seen as an indicator of poor quality of the available remote sensed information.

Clear non-linear patterns emerged with respect to the years of education of the mother, and number of vaccinations. There was a clear non-linear tendency among children whose mothers had up to eight years of schooling having a low height-for-age z-score. For children of mothers with secondary or higher education the height-for-age z-score starts to improve strongly. This trend is consistent with what has been observed in others studies were odds of stunting were higher among children from mothers who had few years of education [12, 52] and lowest among those who had advanced years in education [52]. Higher education level has been associated with increased income levels and improved knowledge among mothers who are usually the primary caregivers. As such educated mothers are more likely to take better care of their children by making informed nutritional decisions [24, 52, 53]. Increasing number of vaccinations showed improved z-score among children. Even though the effect was significant, the same size of the effect might not be relevant in practice.

Moreover, considering the full distribution like we did shows that the variation is highest for low levels of education and decreases with increasing years of education. This study did not consider the association of paternal education and the child z-score, however in another study, it was found that it was Maternal education that had a positive impact on children’s nutritional status [17].

We observe differences in levels of malnutrition in various regions in Zambia. One consistent pattern is that of discrepancy between the rural areas which are worse off compared to urban areas and confirms socio-economic inequalities between rural and urban areas. This may suggest social and economic inequalities between such areas. This has already been documented in other studies [12, 14, 24]. Furthermore, in terms of a spatial distribution, when you consider a smooth spatial effect, there is a clear regional variation in addition to the effect of rurality. Even after accounting for economic activities, the farming southern regions tend to be well off compared to the more industrialised northern areas. There is need to investigate further the underlying factors that contribute to the variation in the height-for-age z-score.

The present study shows that stunting still remain high in Zambia with remarkable regional inequalities and the decline is gradual which is unacceptable. There is need therefore to address the socio-economic indicators if this status is to improve.

## Supporting information

S1 Fig Sampling paths. The Figure depicts the sampling paths of the parameters in *η*_*μ*_ of the effect of the asset index, the two-dimensional effect of the birth order and the age of the mother at birth, the two-dimensional effect of the age of the child and breastfeeding duration, and the effect of the mothers years of education. Demographic and Health Surveys calculation by authors.
(EPS)

S2 Fig Sampling paths. The Figure depicts the sampling paths of the parameters in *η*_*μ*_ of the household size, the linear effects, and the malaria incidence. Demographic and Health Surveys calculation by authors.
(EPS)

S3 Fig Sampling paths. The Figure depicts the sampling paths of the parameters in *η*_*μ*_ of the maternal BMI, the population density, the aridity index, and the spatial effect. Demographic and Health Surveys calculation by authors.
(EPS)

S4 Fig Sampling paths. The Figure depicts the sampling paths of the parameters in *η*_*μ*_ of the effect of the number of vaccination. Demographic and Health Surveys calculation by authors.
(EPS)

S5 Fig Sampling paths. The Figure depicts the sampling paths of the parameters in *η*_*σ*_ of the effect of the asset index, and the effect of the mothers years of education. Demographic and Health Surveys calculation by authors.
(EPS)

S6 Fig Sampling paths. The Figure depicts the sampling paths of the parameters in *η*_*σ*_ of the household size, the linear effects, and the malaria incidence. Demographic and Health Surveys calculation by authors.
(EPS)

S7 Fig Sampling paths. The Figure depicts the sampling paths of the parameters in *η*_*σ*_ of the maternal BMI, the population density, the aridity index, and the spatial effect. Demographic and Health Surveys calculation by authors.
(EPS)

S8 Fig Sampling paths. The Figure depicts the sampling paths of the parameters in *η*_*σ*_ of the effect of the number of vaccination. Demographic and Health Surveys calculation by authors.
(EPS)

## References

[1] World Health Organization. Global nutrition targets 2025: Stunting policy brief. World Health Organization; 2014.

[2] Dwyer-Lindgren L, Kakungu F, Hangoma P, Ng M, Wang H, Flaxman AD, et al. Estimation of district-level under-5 mortality in Zambia using birth history data, 1980–-2010. Spatial and Spatio-temporal Epidemiologyl. 2014;11:89–107. doi: 10.1016/j.sste.2014.09.002 25457599

[3] World Health Organization. The state of food security and nutrition in the world 2018: building climate resilience for food security and nutrition. Food & Agriculture Organization; 2018.

[4] Müller O, Krawinkel M. Malnutrition and health in developing countries. Cmaj. 2005;173(3):279–286. doi: 10.1503/cmaj.050342 16076825 PMC1180662

[5] Black RE, Victora CG, Walker SP, Bhutta ZA, Christian P, de Onis M, et al. Maternal and child undernutrition and overweight in low-income and middle-income countries. The Lancet. 2013;382(9890):427–451. doi: 10.1016/S0140-6736(13)60937-X 23746772

[6] Tette EM, Sifah EK, Nartey ET. Factors affecting malnutrition in children and the uptake of interventions to prevent the condition. BMC pediatrics. 2015;15(1):189. doi: 10.1186/s12887-015-0496-3 26586172 PMC4653928

[7] Menon P, Headey D, Avula R, Nguyen PH. Understanding the geographical burden of stunting in India: A regression-decomposition analysis of district-level data from 2015–16. Maternal & child nutrition. 2018;14(4):e12620. doi: 10.1111/mcn.12620 29797455 PMC6175441

[8] Svedberg P. Undernutrition in Sub-Saharan Africa: Is there a gender bias? The Journal of Development Studies. 1990;26(3):469–486. doi: 10.1080/00220389008422165

[9] Adebayo SB. Modelling childhood malnutrition in Zambia: an adaptive bayesian splines approach. Statistical Methods and Applications. 2003;12(2):227–241. doi: 10.1007/s10260-003-0057-z

[10] Marx S, Phalkey R, Aranda-Jan CB, Profe J, Sauerborn R, Höfle B. Geographic information analysis and web-based geoportals to explore malnutrition in Sub-Saharan Africa: a systematic review of approaches. BMC public health. 2014;14(1):1189. doi: 10.1186/1471-2458-14-1189 25409548 PMC4258026

[11] Gorstein J, Akre J. The Use of Anthropometry to Assess Nutritional Status. World health statistics quarterly Rapport trimestriel de statistiques sanitaires mondiales. 1988;41:48–58. 3176514

[12] Mzumara B, Bwembya P, Halwiindi H, Mugode R, Banda J. Factors associated with stunting among children below five years of age in Zambia: evidence from the 2014 Zambia demographic and health survey. BMC Nutrition. 2018;4(1):51. doi: 10.1186/s40795-018-0260-9 32153912 PMC7050779

[13] UNICEF. Strategy for Improved Nutrition of Children and Women in Developing Countries. A UNICEF Policy Review. New York: UNICEF; 1990.

[14] Black RE, Allen LH, Bhutta ZA, Caulfield LE, de Onis M, Ezzati M, et al. Maternal and child undernutrition: global and regional exposures and health consequences. The Lancet. 2008;371(9608):243–260. doi: 10.1016/S0140-6736(07)61690-0 18207566

[15] UNICEF. Improving child nutrition: the achievable imperative for global progress. New York; 2013.

[16] Gayawan E, Adebayo SB, Komolafe AA, Akomolafe AA. Spatial distribution of malnutrition among children under five in Nigeria: A Bayesian quantile regression approach. Applied Spatial Analysis and Policy. 2019;12(2):229–254. doi: 10.1007/s12061-017-9240-8

[17] Kandala NB, Madungu TP, Emina JB, Nzita KP, Cappuccio FP. Malnutrition among children under the age of five in the Democratic Republic of Congo (DRC): does geographic location matter? BMC public health. 2011;11(1):261. doi: 10.1186/1471-2458-11-261 21518428 PMC3111378

[18] Kandala NB, Fahrmeir L, Klasen S, Priebe J. Geo-additive models of childhood undernutrition in three sub-Saharan African countries. Population, Space and Place. 2009;15(5):461–473. doi: 10.1002/psp.524

[19] Central Statistical Office (CSO)[Zambia] Ministry of Health (MOH)[Zambia] and ICF International. Zambia demographic and health survey 2013–14; 2014.

[20] Ngoma C, Mayimbo S. The Negative Impact of Poverty on the Health of Women and Children. Annals of Medical and Health Sciences Research. 2017;7(6).

[21] Umlauf N, Kneib T. A primer on Bayesian distributional regression. Statistical Modelling. 2018;18(3-4):219–247. doi: 10.1177/1471082X18759140

[22] Kneib T. Beyond mean regression. Statistical Modelling. 2013;13(4):275–303. doi: 10.1177/1471082X13494159

[23] Klein N. An Introduction to Bayesian Structured Additive Distributional Regression; 2013.

[24] Kandala NB, Lang S, Klasen S, Fahrmeir L. Semiparametric Analysis of the Socio-Demographic and Spatial Determinants of Undernutrition in Two African Countries; 2001.

[25] Belitz C, Hübner J, Klasen S, Lang S. In: Determinants of the Socioeconomic and Spatial Pattern of Undernutrition by Sex in India: A Geoadditive Semi-parametric Regression Approach. Heidelberg: Physica-Verlag HD; 2010. p. 155–179.

[26] Brown ME, Grace K, Shively G, Johnson KB, Carroll M. Using satellite remote sensing and household survey data to assess human health and nutrition response to environmental change. Population and environment. 2014;36(1):48–72. doi: 10.1007/s11111-013-0201-0 25132700 PMC4131131

[27] Corsi DJ, Perkins JM, Subramanian S. Child anthropometry data quality from Demographic and Health Surveys, Multiple Indicator Cluster Surveys, and National Nutrition Surveys in the West Central Africa region: are we comparing apples and oranges? Global health action. 2017;10(1):1328185. doi: 10.1080/16549716.2017.1328185 28641057 PMC5496063

[28] Perumal N, Bassani DG, Roth DE. Use and misuse of stunting as a measure of child health. The Journal of nutrition. 2018;148(3):311–315. doi: 10.1093/jn/nxx064 29546307

[29] Hong R, Banta JE, Betancourt JA. Relationship between household wealth inequality and chronic childhood under-nutrition in Bangladesh. International Journal for Equity in Health. 2006;5(1):15. doi: 10.1186/1475-9276-5-15 17147798 PMC1702347

[30] Filmer D, Pritchett LH. Estimating Wealth Effects Without Expenditure Data—Or Tears: An Application To Educational Enrollments In States Of India. Demography. 2001;38(1):115–132. doi: 10.1353/dem.2001.0003 11227840

[31] Sahn DE, Stifel D. Exploring Alternative Measures of Welfare in the Absence of Expenditure Data. Review of Income and Wealth. 2003;49(4):463–489. doi: 10.1111/j.0034-6586.2003.00100.x

[32] Frongillo Edward A Jr, de Onis M, Hanson KMP. Socioeconomic and Demographic Factors Are Associated with Worldwide Patterns of Stunting and Wasting of Children. The Journal of Nutrition. 1997;127(12):2302–2309. doi: 10.1093/jn/127.12.2302 9405578

[33] Osgood-Zimmerman A, Millear AI, Stubbs RW, Shields C, Pickering BV, Earl L, et al. Mapping child growth failure in Africa between 2000 and 2015. Nature. 2018;555:41–47. doi: 10.1038/nature25760 29493591 PMC6346257

[34] Amoah B, Giorgi E, Heyes DJ, van Burren S, Diggle PJ. Geostatistical modelling of the association between malaria and child growth in Africa. International Journal of Health Geographics. 2018;17(1):7. doi: 10.1186/s12942-018-0127-y 29482559 PMC5828493

[35] Bhatt S, Weiss DJ, Cameron E, Bisanzio D, Mappin B, Dalrymple U, et al. The effect of malaria control on Plasmodium falciparum in Africa between 2000 and 2015. Nature. 2015;526:207–211. doi: 10.1038/nature15535 26375008 PMC4820050

[36] van der Schrier G, Barichivich J, Briffa KR, Jones PD. A scPDSI-based global data set of dry and wet spells for 1901–2009. Journal of Geophysical Research: Atmospheres. 2013;118(10):4025–4048.

[37] Barichivich J, Osborn TJ, Harris I, van der Schrier G, Jones PD. Drought. In: Hartfield G, Blunden J, Arndt DS, editors. State of the Climate in 2018. vol. 100. Bulletin of the American Meteorological Society; 2018.

[38] Center for International Earth Science Information Network—CIESIN—Columbia University. Gridded Population of the World, Version 4 (GPWv4): Population Density, Revision 10; 2017.

[39] Adebayo SB, Fahrmeir L. Analysing child mortality in Nigeria with geoadditive discrete-time survival models. Statistics in Medicine. 2005;24(5):709–728. doi: 10.1002/sim.1842 15696506

[40] Osei FB, Duker AA, Stein A. Bayesian structured additive regression modeling of epidemic data: application to cholera. BMC Medical Research Methodology. 2012;12(1):118. doi: 10.1186/1471-2288-12-118 22866662 PMC3528434

[41] Klein N, Kneib T, Klasen S, Lang S. Bayesian structured additive distributional regression for multivariate responses. Journal of the Royal Statistical Society: Series C (Applied Statistics). 2015;64(4):569–591. doi: 10.1111/rssc.12090

[42] Seiler J, Harttgen K, Kneib T, Lang S. Modelling children’s anthropometric status using Bayesian distributional regression merging socio-economic and remote sensed data from South Asia and sub-Saharan Africa. Economics & Human Biology. 2021;40:100950. doi: 10.1016/j.ehb.2020.100950 33321408

[43] Klein N, Kneib T, Lang S, Sohn A. Bayesian structured additive distributional regression with an application to regional income inequality in Germany. Ann Appl Stat. 2015;9:1024–1052. doi: 10.1214/15-AOAS823

[44] Dunn PK, Smyth GK. Randomized Quantile Residuals. Journal of Computational and Graphical Statistics. 1996;5(3):236–244. doi: 10.1080/10618600.1996.10474708

[45] Belitz C, Brezger A, Klein N, Kneib T, Lang S, Umlauf N. BayesX: Software for Bayesian Inference in Structured Additive Regression Models. Version 3.0.2; 2015. Available from: http://www.BayesX.org/.

[46] Lang S, Brezger A. Bayesian P-Splines. Journal of Computational and Graphical Statistics. 2004;13(1):183–212. doi: 10.1198/1061860043010

[47] Eilers PHC, Marx BD. Flexible smoothing with B-splines and penalties. Statististical Science. 1996;11(2):89–121. doi: 10.1214/ss/1038425655

[48] Hastie TJ, Tibshirani RJ. Varying-Coefficient Models. Journal of the Royal Statistical Society Series B (Methodological). 1993;55(4):757–796. doi: 10.1111/j.2517-6161.1993.tb01939.x

[49] Fahrmeir L, Kneib T, Lang S, Marx B. Regression: Models, Methods and Applications. 1st ed. Springer-Verlag Berlin Heidelberg; 2013.

[50] Spiegelhalter DJ, Best NG, Carlin BP, Van der Linde A. Bayesian Measures of Model Complexity and Fit. Journal of the Royal Statistical Society Series B (Statistical Methodology). 2002;64(4):583–639. doi: 10.1111/1467-9868.00353

[51] Watanabe S. Asymptotic equivalence of Bayes cross validation and widely applicable information criterion in singular learning theory. Journal of Machine Learning Research. 2010;11:3571–3594.

[52] Angdembe MR, Dulal BP, Bhattarai K, Karn S. Trends and predictors of inequality in childhood stunting in Nepal from 1996 to 2016. International journal for equity in health. 2019;18(1):42. doi: 10.1186/s12939-019-0944-z 30836975 PMC6402091

[53] Svefors P, Rahman A, Ekström EC, Khan AI, Lindström E, Persson LÅke, et al. Stunted at 10 years. Linear growth trajectories and stunting from birth to pre-adolescence in a rural Bangladeshi cohort. PloS one. 2016;11(3):e0149700. doi: 10.1371/journal.pone.0149700 26934484 PMC4775024

[54] Hijmans, Robert J, University of California, Berkeley, Museum of Vertebrate Zoology. Global Administrative Areas (GADM), version 3.6; 2018. Available from: http://gadm.org.

